# UCP2, a Member of the Mitochondrial Uncoupling Proteins: An Overview from Physiological to Pathological Roles

**DOI:** 10.3390/biomedicines12061307

**Published:** 2024-06-13

**Authors:** Salvatore Nesci, Speranza Rubattu

**Affiliations:** 1 Department of Veterinary Medical Sciences, Alma Mater Studiorum University of Bologna, 40064 Ozzano Emilia, Italy; salvatore.nesci@unibo.it; 2 Department of Clinical and Molecular Medicine, School of Medicine and Psychology, “Sapienza” University of Rome, 00189 Rome, Italy; 3 IRCCS Neuromed, 86077 Pozzilli, Italy

**Keywords:** bioenergetics, mitochondria, H^+^ leak, UCP2, oxidative phosphorylation, cardiovascular diseases

## Abstract

UCP2 is an uncoupling protein homolog to UCP1. Unlike UCP1, which participates in non-shivering thermogenesis by uncoupling oxidative phosphorylation (OXPHOS), UCP2 does not perform a canonical H^+^ leak, consuming the protonmotive force (Δ*p*) through the inner mitochondrial membrane. The UCP2 biological role is elusive. It can counteract oxidative stress, acting with a “mild uncoupling” process to reduce ROS production, and, in fact, UCP2 activities are related to inflammatory processes, triggering pathological conditions. However, the Δ*p* dissipation by UCP2 activity reduces the mitochondrial ATP production and rewires the bioenergetic metabolism of the cells. In all likelihood, UCP2 works as a carrier of metabolites with four carbon atoms (C4), reversing the anaerobic glycolysis-dependent catabolism to OXPHOS. Indeed, UCP2 can perform catalysis in dual mode: mild uncoupling of OXPHOS and metabolite C4 exchange of mitochondria. In vivo, the UCP2 features in the biology of mitochondria promote healthy ageing, increased lifespan, and can assure cerebro- and cardiovascular protection. However, the pathological conditions responsible for insulin secretion suppression are dependent on UCP2 activity. On balance, the uncertain biochemical mechanisms dependent on UCP2 do not allow us to depict the protective role in mitochondrial bioenergetics.

## 1. Introduction

The energy transduction mechanism in mitochondria performs the conversion of the chemical potential of substrate oxidation in protonmotive force (Δ*p*) to drive ADP phosphorylation. Mitochondrial respiratory complexes exploit the redox potential of NADH- or FADH_2_-linked reducing equivalents, working as H^+^ pumps, and establish the Δ*p* through the inner mitochondrial membrane (IMM). The Δ*p* dissipated by F_1_F_O_-ATPase is transformed in the energy chemical bond with ADP phosphorylation to ATP [1]. The energy conversion in this mitochondrial chemiosmotic H^+^ circuit is fulfilled in the oxidative phosphorylation (OXPHOS) system. Therefore, the efficiency of the process relies on the capacity of coupling oxygen consumption to ADP phosphorylation. Otherwise, pathological or physiological conditions can hinder OXPHOS [2,3]. In particular, the Δ*p* de-coupled from ATP synthesis might be affected by specific proteins in the IMM, impairing the OXPHOS [4] via different mechanisms of energy consumption.

## 2. The Uncoupling of OXPHOS

**Figure 1:**
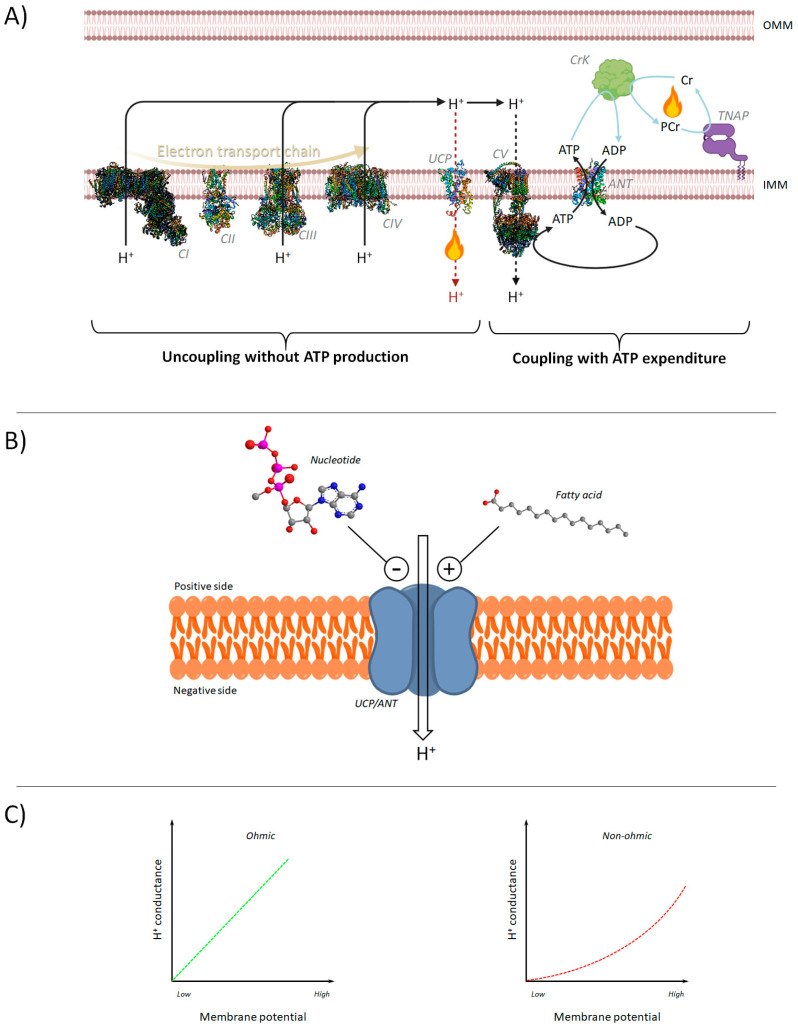
Mitochondrial bioenergetic features. (**A**) Schematic representation of protonmotive force by uncoupling driven by UCP or ATP-dependent futile cycle. CI, CII, CIII, and CIV identify the respiratory complexes, whereas CV is the F_1_F_O_-ATPase. ANT, adenine nucleotide translocase; CrK, creatine kinase, TNAP, tissue-nonspecific alkaline phosphatase; IMM and OMM, inner and outer mitochondrial membrane, respectively. (**B**) Positive and negative regulation of H^+^ leak by carrier. (**C**) Relationship of H^+^ conductance vs. membrane potential for ohmic (on the **right**) and non-ohmic (on the **left**) circuits.

Uncoupling protein-1 (UCP1) is the protein responsible for producing heat in the mitochondria of brown adipose tissue. Working as a proton carrier, UCP1 catalyzes an H^+^ leak, consuming the Δ*p* built at the expense of the energy of substrates oxidized on respiration. This physiological uncoupling of UCP1 can mediate adaptive non-shivering thermogenesis, increasing the energy expenditure [5,6]. The H^+^ transport mediated by UCP1 needs free fatty acids, whereas it is inhibited by nucleotides such as GTP [7]. The physiological differences between thermogenesis supported by UCP1 and UCP1-independent thermogenesis, through ATP-consuming futile cycle in mitochondria or between multiple subcellular compartments, consist of Δ*p* dissipation with two different mitochondrial mechanisms of uncoupling, without ATP production or coupling with ATP expenditure, respectively [4,8]. In the latter bioenergetic phenomenon, the Δ*p* is dissipated by F_1_F_O_-ATPase as in the normal function of OXPHOS, but the synthesized ATP is not used in cellular processes [9]. Indeed, in the mitochondrial creatine-dependent ATP/ADP substrate cycling, the phosphocreatine (PCr)/creatine (Cr) kinase system hydrolyzes the mitochondrial ATP to ADP, and the Cr is phosphorylated to PCr. Then, tissue-nonspecific alkaline phosphatase hydrolyses PCr to initiate a futile cycle of Cr phosphorylation and dephosphorylation by consuming ATP [10] (Figure 1A). Noteworthy are the energy-dissipating mechanisms of ATP-consuming cycles that connect multiple subcellular compartments and/or tissues. Indeed, the calcium cycle causes ineffective ATP consumption, which is responsible for energy dissipation. The endoplasmic reticulum (ER) of beige adipocytes promotes the efflux of Ca^2+^ in response to cold exposure driven by norepinephrine-mediated stimulation of ryanodine receptor 2 (RyR2) activity on ER. The accumulation of cytosolic Ca^2+^ stimulates its uptake by the sarcoendoplasmic reticulum Ca^2+^, known as SERCA, which mediates calcium cycling, sustained by ATP synthetized in mitochondria. Ca^2+^ exported in the cytosol by RyR2 is imported into the ER by the Ca^2+^-ATPase activity of SERCA. This ATP-dependent futile cycle is a thermogenic mechanism used by beige adipocytes expressing a high level of F_1_F_O_-ATPase to produce heat, dissipating the Δ*p* and regulating glucose homeostasis [11]. Moreover, the glycerol *de novo* synthesis by glyceroneogenesis and coenzyme A addition to free fatty acids, which are necessary for promoting the triglyceride re-esterification in adipose tissue, causes a futile ATP consumption by the lipid cycle of fatty acids storage and triacylglycerols degradation [9].

Other proteins and biophysical conditions can cause H^+^ leak across the IMM [4]. Indeed, together with UCP1, the adenine nucleotide translocase (ANT), also called ADP/ATP transporter (AAC), can mediate fatty acid-dependent H^+^ transport [12] using lipid molecules as co-factors to support the translocation of protons. One of the primary physiological sources of free fatty acid production might rely on the activity of phospholipase A2 [13], which could be responsible for ANT-dependent H^+^ leak. In general, UCP1 expressed in brown/beige adipose tissue is responsible for H^+^ leak, whereas this inducible H^+^ conductance across the IMM depends on ANT in all other tissues. The primary common features of H^+^ leak mediated by ANT and UCP1 are fatty acid-activated and purine nucleotide negative-regulated [14] (Figure 1B). However, a known non-inducible “basal H^+^ leak” across the lipid bilayer, due to non-specific IMM permeability to H^+^, violates Ohm’s Law, exponentially increasing at high values of membrane potential [4,15] (Figure 1C).

It could be interesting to consider the transhydrogenation of electrons transferred by nicotinamide nucleotide transhydrogenase (NNT) from NADH to reduce NADP^+^ to NADPH [16]. The Δ*p* dissipated by NNT-mediated H^+^ translocation generates NADPH in an energy-consumed mechanism not linked to thermogenesis. However, the NNT uncoupling of mitochondrial respiration from ATP synthesis sustains the antioxidant defence and sirtuin actions of mitochondria [17].

Other UCPs proteins, i.e., uncoupling protein-2 (UCP2) and uncoupling protein-3 (UCP3), might have physiological roles different from the obvious thermogenic functions explained above and attained through the respiratory uncoupling phenomenon. Oxidative stress and lipotoxicity in mitochondria inducing metabolic diseases and energy unbalance of metabolic process [2] could be counteracted by UCP activity. UCP2 is identified in a variety of organs, the kidney in particular, whereas UCP3 is primarily encountered in skeletal muscle. However, the story of the putative new uncoupling proteins, UCP2 and UCP3, is a little more complicated [18]. Indeed, there are two hypotheses: either they work as functional protonophoric homologues of UCP1 or the proteins are conventional metabolite transporters. Similar to UCP1, the photonophoric regulatory mechanism of UCP2 and UCP3 might rely on high sensitivity to fatty acid activation and inhibition exerted by purine nucleotide. The physiological role of the UCP1-like mechanism has led to the concept of “mild uncoupling” not related to thermogenesis but as a protective mechanism against oxidative stress [18,19].

In the absence of nucleotides, UCP3 catalyzes remarkable GDP-sensitive and superoxide-stimulated protonophoric activity. The mild uncoupling promoted by UCP3 is superoxide-dependent and plays an important role in controlling ROS production. However, superoxide does not directly activate UCP3, but it acts indirectly through the hydroxynonenal (HNE) generated with lipid peroxidation [20]. Alternative suggestions for the functions of uncoupling proteins consider pyruvate and amino acid metabolism [21,22].

In this review, the multifaceted role of UCP2 in physiological and pathological conditions will be considered in light of increasing evidence of the protein function(s) and the elucidation of the underlying mechanisms in mitochondria bioenergetics.

## 3. UCP2: A Possible Multifunctional Mitochondrial Carrier

UCP2 participates in various physiological and pathological processes, but its biological role is still elusive (Table 1). The UCP2 biochemical and physiological functions can cover the role of protecting cells from oxidative stress; increasing antitumor immunity in the tumour microenvironment to limit cancer development; regulating tumour progression through changes in glycolytic, oxidative, and calcium metabolism [23]; defending target organs in cardiovascular diseases such as hypertension [24]. Rising ionic leaks in the IMM are known as mitochondrial respiration dissociated from ATP synthesis, leading to uncoupling. UCP2 can affect the OXPHOS system by decreasing the membrane potential [25]. However, the H^+^ leak associated with UCP2 could be related to a thermogenic phenotype of UCP1 generated from the uncoupling of OXPHOS [26]. The UCP2 activity is increased in the presence of ROS in a manner dependent on fatty acid oxidation. As a result, the UCP2 acts to revert ROS production by decreasing the membrane potential of mitochondria through a mechanism of H^+^ leak that could be different from UCP1. Indeed, essential histidine residues of UCP1 supporting the protonophore kinetics of the enzyme are absent in UCP2 [27].

The UCP2 aids mitochondrial functions by constraining excessive ROS production with the “mild uncoupling” process. Otherwise, it can cause an unbalance in mitochondrial ATP production by increasing the ADP/ATP ratio. In particular, H^+^ leak catalyzed by UCP2, bypassing the F_1_F_O_-ATPase, reduces the cellular ATP content in pancreatic β cells. The decrease in insulin secretion is related to the decline in exocytosis of insulin vesicles in glucose-stimulated insulin secretion conditions. The molecular mechanism is explained by the polarization of the plasma membrane with the ATP-gated K^+^ channel opened under a low level of ATP, which, in turn, closes the voltage-gated Ca^2+^ channel. The missing energetic signalling of mitochondrial ATP production, eluded by UCP2 activity, blocks the translocation of insulin granules and release [41].

Insulin resistance develops from chronic inflammation of lipids in adipose tissue, particularly in patients with metabolic syndrome or in obese subjects [42]. The altered microenvironment of adipose tissue causes customised metabolic reprogramming, which promotes tissue inflammation. UCP2 deficiency dramatically enhances glycolysis and oxidative respiration in the setting of obesity-induced adipose tissue inflammation and insulin resistance. However, UCP2 activity does not perform an important role in shaping macrophage activation in adipose tissue of diabetes associated with insulin resistance [43]. Thus, in inflammatory or autoimmune diseases, UCP2 can control macrophage activation by modulating the production of mitochondrial ROS [44].

A reduction in the UCP2 mRNA level was found to be related to a consequent increase in ROS generation. Thus an increase in the UCP2 mRNA level was associated with reduced ROS production [45]. Indeed, UCP2 deletion increased macrophage formation of ROS, highlighting the hypothesis that UCP2 could be triggered by superoxide, providing negative feedback to limit oxidative damage [18]. The UCP2 function linked to cellular metabolism affects the redox state and the oxidative stress of the cells. Interestingly, in this context, UCP2 plays a role in chronic inflammation associated with related disorders of atherogenesis and low-density lipoprotein oxidation as a consequence of obesity [46]. Atherogenesis triggered by inflamed endothelial cells is controlled by stress-dependent regulation of UCP2 expression by Krüppel-like factor 2. Increased UCP2 levels produce a mechanosensitive action, which leads to decreased inflammation. In this condition, a reduced Western diet-induced atherosclerosis and less-disturbed flow-associated atherosclerosis are observed [28]. The role of UCP2 as a regulator of mitochondrial ROS production is corroborated by results in the presence of nucleotide GDP that blocks the UCP2 activity, causing mitochondrial membrane polarization and ROS production [46].

Mild uncoupling of OXPHOS, reducing mitochondrial ROS production, avoids mitochondrial dysfunction. The products of lipid oxidative stress, e.g., HNE, *trans*-retinoic acid, *trans*-retinal, and other reactive 2-alkenal groups, lead to the uncoupling of mitochondria, stimulating the UCPs. In kidney mitochondria, ROS production-induced ROS release [47] is decreased by UCP2. However, GDP-sensitive ROS released under HNE conditions is partially hampered [48].

Hepatocytes can accumulate fatty acids, and liver lipotoxicity induces metabolic-associated fatty liver disease (MAFLD). UCP1 is not present in any liver cell type. Conversely, UCP2 is expressed in Kupffer cell-specific macrophages of the liver [49]. Immunomodulation of the MAFLD linked to the inflammatory response in macrophage mitochondria could be related to the development of fibrosis, which triggers steatohepatitis or dangerous conditions of cirrhosis and hepatocarcinoma [50].

Moreover, in cancer, UCP2 could allow for ROS accumulation during the initial phase of tumour growth. Indeed, controlled elevated levels of ROS promote tumour growth by acquiring oncogene mutations and activating pro-tumour metabolic signals related to the depletion of antioxidant systems [51]. ROS control in tumour cells could be restrained by UCP2. UCP2 deficiency might promote tumour cells that are less likely to metastasize. Therefore, UCP2 as a metabolic sensor can reprogram the tumour metabolism of cells, with metabolites exported by UCP2 reversing the Warburg effect to oxidative phosphorylation. The decrease in glycolysis-dependent metabolism can reduce the tumorigenic capacity of cells [29]. However, there is evidence suggesting that UCP2 involvement in cancer cell glycolytic metabolism has a poor prognosis on tumour growth, relying on a different energy metabolism [30]. In particular, UCP2 can be involved in two events related to the Warburg effect, promoting metabolic adaptations of cancer cells. UCP2 can reduce the redox pressure on the mitochondrial respiration by exporting metabolites of tricarboxylic acid cycle (TCA) and, as a consequence, prevent ROS generation; stimulate glutamine oxidation as an energetic fuel [31]. Depending on whether tumor metabolism is oxidative or glycolytic, the transporter role of UCP2 in cancer may have a distinct effect on the development of cancer [32,33].

By considering the regulatory role of ROS production in mitochondria, alternative suggestions on functional features of UCP2 have included glutathione transport as a mechanism for resistance to cerebral ischemic injury. UCP2 mRNA is massively induced after three days post-ischemia, which provides a decreased resistance to cerebral ischemia due to an increased oxidative injury and downed cerebral neuronal antioxidant condition. Therefore, the elimination of ROS might be accomplished with glutathione in the reduced state in the matrix [34]. UCP2 may perform other functions related to cellular metabolism. Genetic loss of UCP2 induces the stimulation of glucose metabolism. By promoting a shift from sugar to fatty acids metabolism, UCP2 transports the fatty acids out of mitochondria, hindering oxidation [52].

Together with glucose, glutamine is an essential oxidative fuel in immune and proliferative cells, and both are preferentially consumed by fast-growth cells to the detriment of mitochondrial sources of energy [53]. The absence of UCP2 is associated with decreased glutaminolysis in mitochondria [54].

The dual mode work of UCP2 might consider the uncoupling mechanism responsible for ROS regulation or substrate exchange transport that might deplete the mitochondria of intermediate compounds of the TCA. In both cases, UCP2 can repress ATP-producing reactions, driving mitochondria towards the non-phosphorylating state. As a consequence, a cellular adaptation to a reduced substrate or oxygen supply is induced. In particular, under ischemic conditions, the adenine nucleotide pool decreases, whereas succinate accumulates in mitochondria [55]. After reperfusion, the excess of succinate is oxidized, sustaining the hyperpolarization of IMM by canonical electron transfer through complex III and complex IV to oxygen and maintaining the reduced coenzyme Q pool. The latter, sustained by the membrane potential, drives the reverse electron transfer at complex I to produce ROS responsible for the ischemia–reperfusion (IR) injury. Protection against IR injury is obtained with rotenone or malonate, which block the oxidation of coenzyme Q and succinate at complex I and complex II sites, respectively [56,57]; by mild uncoupling dissipating the Δ*p* [58]. As a result, UCP2 activity could establish cellular conditions of resistance to pathological situations [59].

In a contest of promotion of healthy ageing and increased lifespan, the cell metabolism might be boosted by a UCP2-dependent longevity activity. The maintenance of fatty acid oxidation and restriction of subsequent oxidative damage related to lipid catabolism allow for to maintenance of the mitochondrial oxidative capacity and biogenesis [60]. The involvement of UCP2 functions in the maintenance of mitochondrial activity, exploiting the control of oxidative stress in physiological or pathological conditions, can affect the immune response and inflammation, thus characterizing the septic acute kidney injury [35]. ROS production causes mitochondrial dysfunction and, in turn, the oxidation of mitochondrial DNA (mtDNA). Damaged mtDNA is cleaved and exported out mitochondria through the mitochondrial permeability transition pore, an ion non-selective membrane pore involved in a wide range of mitochondrial dysfunction events, triggering regulated cell death, and the voltage-dependent anion channel. These two different pores are placed on the inner and outer mitochondrial membrane, respectively. The mtDNA released in the cytosol acts as a DAMP (damage-associated molecular pattern) molecule, activating the inflammasome and the cGAS-STING complex responsible for proinflammatory molecules, such as interleukin-1β and interleukin-18 and interferon signalling [61,62]. Thus, the antioxidant activities of UCP2 can revert the renal injury aggravated by lipopolysaccharide (LPS), counteracting the inflammation, macrophage infiltration, and the mitochondrial dysfunction linked to oxidative stress [35].

In addition to the protection against ROS formation, the physiological functions of UCP2 have been revised to include obesity protection and thermogenic involvement in the fever response. In the latter case, LPS stimulation, which induces fever, causes a remarkable increase in the mRNA and protein levels of UCP2 in different tissues, including the lung [63], brain [64], and liver [65,66].

An increasing amount of evidence, as detailed below, suggests that UCP2 should be classified as a mitochondrial transporter rather than an uncoupling protein.

## 4. Mitochondrial Respiration and the Role of UCP2 as Uncoupling Protein or Metabolite Carrier

**Figure 2:**
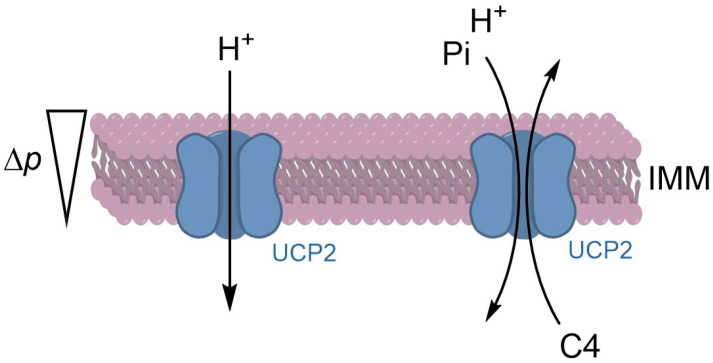
Proposed mechanism of uncoupling protein-2 (UCP2)-mediated transport activity. The mild uncoupling by H^+^ leak acting as a relief valve on polarized IMM is depicted on the left. UCP2 transport of C4 metabolites is shown on the right. Δ*p*, proton motive force; C4, metabolites with four carbon atoms; Pi, inorganic phosphate; IMM, inner mitochondrial membrane.

UCP2 expression under conditions of stimulated fatty acid metabolism may be related to an as yet unidentified role in the metabolism of lipid molecules. Indeed, UCP2 is a *bona fide* uncoupling protein that decouples the OXPHOS, but it should rather be considered as a mitochondrial metabolite carrier [52]. Since UCP2 is present in fish, it is possible to speculate that this carrier is not directly associated with homeothermia, endothermia, non-shivering thermogenesis, or high mammalian basal metabolic rate [67]. We can assert that the H^+^ leak activity of UCP2 is a “relief valve” for the polarized IMM (Figure 2) to avoid the formation of superoxide anion in conditions in which the mitochondrial electron carriers of the respiratory chain are in a reduced state [4,68].

UCP2 is not present in all organisms alive today, even if it is evolutionarily ancient. It is considered a member of the larger family of mitochondrial anion carrier proteins identified as SLC25. The transporters of this family ensure the supply of ions, important metabolic intermediates, and coenzymes to mitochondria. UCP2 might facilitate the transfer of anions from the negative to the positive side of IMM and the return transfer of H^+^ from the positive to the negative side of IMM [69]. Considering the high homology between UCP1 and UCP2, researchers initially focused on the similar mitochondrial membrane uncoupling of OXPHOS. These assumptions are also consistent with the results of Rial et al., who argue that purine nucleotides, palmitic acid, and retinoic acid do not influence the respiratory rate of UCP2-overexpressing yeast mitochondria under standard conditions (pH 6.8) [70]. In contrast, they discovered that an increase in pH is positively regulated by retinoids, increasing mitochondrial respiration. It has been shown that pHs ranging from 6.8 to 7.5 and retinoic acid are two parameters that can increase the uncoupling activity of UCP2 [70]. Indeed, the first study on UCP2 KO mice showed that UCP2 has no uncoupling function at physiological levels since the deletion of UCP2 does not change spleen mitochondrial respiration [71].

The uptake of Ca^2+^ in mitochondria is performed by macromolecular complexes that consist of the pore-forming mitochondrial Ca^2+^ uniporter (MCU) protein, the essential MCU regulator (EMRE), the mitochondrial Ca^2+^ uptake 1 (MICU1), and MICU2 in the intermembrane space [72]. Methylation of Arg_455_ of MICU1 by protein arginine methyl transferase 1 (PRMT1) is a post-translational modification that desensitizes the mitochondrial Ca^2+^ uniporter complex for Ca^2+^, decreasing the uptake of the divalent cation. In various age-related diseases, such as cancer, neurodegenerative, and metabolic/cardiovascular disorders, the PRMT1 activity increases, impairing mitochondrial Ca^2+^ homeostasis [73]. UCP2 binds primarily to the methylated MICU1, restoring Ca^2+^ sensitivity and mitochondrial Ca^2+^ uptake. Therefore, under situations of enhanced PRMT1 activity, UCP2 is described as a sensitizer of Ca^2+^ uniport in mitochondria, acting as a unique regulator of methylated MICU1 [74].

UCP2 can regulate the substrate oxidation in mitochondria, supporting the transport of metabolites with four carbon atoms (C4) and playing a role in reprogramming metabolic pathways. The metabolic switch from fatty acid oxidation to glucose metabolism is supported in UCP2^−/−^ proliferating cells [75]. The transport mechanism of UCP2 is catalyzed by an exchange of cytosolic phosphate inorganic (Pi) for intramitochondrial C4 metabolites. The antiport mechanism of Pi/C4 is conducted by the H^+^ uptake in cotransport with Pi. In other words, Pi and H^+^ are uptaken in the matrix, whereas the C4 is moved out of mitochondria (Figure 2). The H^+^-assisted mechanism is driven by IMM membrane potential (negative inside) and pH gradient (acidic outside) of energized mitochondria [54].

However, the exchange mechanism of the carrier can explain the bioenergetic role of UCP2 for the mild uncoupling, the metabolic reprogramming under different physiological or pathological conditions as well as the tissue-specific distribution and the common presence in endotherms and in the ectotherm kingdom, where thermogenesis is absent. Indeed, oxalacetate is a C4 compound in mitochondria exported outside by UCP2. The TCA cycle starts with the citrate synthesis, driven by the condensation reaction of acetyl-CoA to oxaloacetate. The availability of the latter limits the cycle reactions of the pathway. The UCP2 catalyzing the oxalacetate transport decreases the production of NADH and FADH_2_ in the TCA cycle, limiting the electron flux and lowering the redox pressure on the mitochondrial respiration. As a consequence, the attenuation of mitochondrial respiration reduces the ATP synthesis by F_1_F_O_-ATPase and the ROS generation [54]. These conditions stimulate aerobic glycolysis, hindering mitochondrial glucose oxidation [76]. In β cells, the oxalacetate transport by UCP2 can explain the restraint of insulin secretion, caused by the increased ADP/ATP ratio and lactate release [77].

Glutaminolysis is an anaplerotic pathway used to fuel the TCA cycle in proliferating cells [53]. The glutamine transformation restores oxaloacetate for continued TCA cycle function (anaplerosis) required for rapid synthesis of macromolecules, including lipids, proteins, and nucleotides [78], skipping the OXPHOS in the metabolic adaptations required for cell proliferation. UCP2 expression is stimulated by glutamine, and the flux of glutaminolysis decreases in the absence of a carrier [79]. Moreover, the UCP2-mediated export of C4 compounds can promote the Warburg effect by shifting glucose utilization from respiration to anabolic reactions for macromolecule synthesis. Thus, UCP2 is implicated in two Warburg effect-related events: the utilization of glutamine as an energetic fuel and the export of TCA cycle C4 metabolites from mitochondria for macromolecule biosynthesis [31]. Indeed, UCP2 silencing in human lung adenocarcinoma cell line A549, which is actively proliferating, lowers basal respiration. The inhibition of F_1_F_O_-ATPase synthesis of ATP is not compensated by an increase in glycolysis. Thus, the tumour cells might rely on reduced cellular ATP demand, insensitive to an impaired bioenergetic metabolism, but it depends on a metabolic adaptation of proliferating cells supported by C4-metabolite transported by UCP2 [31].

Therefore, the role of UCP2 as a metabolic sensor contributes to lipid metabolism regulation by supporting a switch from carbohydrate to lipid metabolism. In the absence of UCP2, pyruvate accumulation in the matrix could provide an adequate supply to the TCA cycle that inhibits the fatty acids and glutamine oxidation [80]. However, the role of efflux of pyruvate is physiologically linked with UCP2 tissue localization and expression.

## 5. Pathogenetic Role of UCP2 Deficiency in Animal Models and Humans

The cytoprotective effects of UCP2 were extended to animal and human tissues/organs in both physiological and pathological conditions. Since UCP2 supports fatty acid oxidation and mainly limits oxidative damage, allowing for a regular mitochondrial oxidative capacity and mitochondrial biogenesis, it can promote healthy ageing and increased lifespan. The metabolic hypothesis of UCP2-dependent longevity was proven in transgene and *knock-out* mice models: UCP2 transgene revealed an extended lifespan, whereas mice deficient in UCP2 showed a significantly shorter lifespan [81]. A decrease in UCP2 was reported in isolated cardiomyocytes from old mice compared to young ones [82] and also in cardiac tissue-derived stem-like cells (CTSCs). In the latter experimental condition, UCP2 was highly expressed in CTSCs from the neonatal heart. In contrast, UCP2 was reduced in aged CTSCs, shifting energy metabolism to OXPHOS, thus reducing cellular proliferation and survival [83]. In line with this evidence, it was recently demonstrated that UCP2-KO mice kept under moderate hypoxia showed a more pronounced decline in cardiac function compared to *wild-type* (*wt*) mice. In addition, cardiomyocyte cell cycle activity was reduced, while fibrosis and DNA damage were significantly increased in UCP2KO compared with *wt* under hypoxia. The results support a role for mitochondrial UCP2 as an oxygen sensor, regulating cardiomyocyte cell cycle activity, acetyl-CoA levels, and histone acetylation in response to moderate hypoxia [84]. In a different animal model, the spontaneously hypertensive rat showing a higher predisposition to target organ damage development (SHRSP), an age-related decrease in UCP2 expression, was found in the heart, kidney, and microglia compared to its strictly related control, the stroke-resistant SHR (SHRSR) [36].

However, a protective effect of UCP2 has been reported in both cerebrovascular and ischemic heart disease. UCP2 protects the heart from IR injury, being upregulated in this condition along with increased autophagy stimulation [37]. Two experimental models of stroke revealed the neuroprotective effect of UCP2: the MCAO mouse [85] and the SHRSP [86]. The experience gained with SHRSP investigation strongly supported a deleterious consequence of a stroke-promoting diet on UCP2 expression in the brain of SHRSP vs. SHRSR. The decrease in UCP2 induced by the high-salt diet, which is mediated by microRNA-503, involved an impaired mitochondrial function with increased ROS accumulation. In this context, the significant therapeutic effect of a few approaches able to stimulate UCP2 expression, fenofibrate and a *Brassica oleracea sprout* extract, was revealed [86]. An intriguing interplay between salt loading, ROS, UCP2, and autophagy was later discovered [87]. In humans, UCP2 and UCP5 expression was significantly increased in brain ischemic lesions compared to the intact area. This evidence could be interpreted as an adaptive protective response to the ischemic insult [88].

UCP2 is involved in obesity and diabetes mellitus, mainly through its ability to suppress insulin secretion [6,39]. On the other hand, UCP2 downregulation favoured insulin secretion and obesity [39]. Although UCP2 is a mitochondrial protein that inhibits insulin secretion from β cells, it is unaffected by excessive glucosamine exposure, in both pancreatic islets and the β-cell line [89]. Instead, genipin, an inhibitor of UCP2, can reduce the expression of UCP2, limiting the amount of glucose that can be transferred to cells and lowering the production of advanced glycation end products (AGEs) and receptor for AGEs [90]. UCP2 mutations are associated with human diseases, highlighting an important role for mitochondria in congenital hyperisulinism [38]. In a rat model of metabolic syndrome, the increase in UCP2 in the liver serves as a mechanism of protection toward oxidative stress [9,91]. Accordingly, UCP2 stimulation through a dietary intervention in this model improved lipid metabolism and mitochondrial function along with an increase in UCP2 expression in several tissues [10,92]. Since UCP2 influences ROS generation, cell metabolism and proliferation, apoptosis and autophagy, its role was also explored in cancer and tumour drug sensitivity. As a result, a controversial role of UCP2 has been reported in these conditions [93].

Although a clear pathogenic effect of UCP2 has not been demonstrated yet in human diseases, an interesting variant of the human gene (-866G>A SNP), with the A allele being associated with increased UCP2 expression and decreased ROS accumulation, was shown to associate with obesity and diabetes in several populations [40].

## 6. Conclusions

Based on the available experimental evidence, UCP2 emerges as a mitochondrial protein able to exert important modulatory effects in both physiology and pathology in several organs and apparatuses. It is even expected that the modulation of UCP2 expression may serve as a suitable pharmacological strategy to treat common human diseases. However, the unsolved biochemical mechanism of the enzymatic catalysis of UCP2 makes it difficult to attribute a definite cellular role. Recent evidence argues that UCP2 is a C4 metabolite rather than an uncoupling carrier. The elucidation of this arcane phenomenon could explain the physiological or pathological behaviour of UCP2 in mitochondrial bioenergetics.

## Figures and Tables

**Table 1:** Deleterious vs. beneficial roles of UCP2 in different pathological conditions.

Diseases	UCP2 Effect on Health (−) Deleterious/(+) Beneficial	References
Atherogenesis	+	[28]
Melanoma	+	[29]
Cholangiocarcinoma	−	[30]
Lung adenocarcinoma	−	[31]
T-cell acute lymphoblastic leukemia	−	[32]
Colorectal cancer	+	[33]
Cerebral ischemic injury	−	[34]
Septic acute kidney injury	+	[35]
Hypertension	+	[36]
Cerebrovascular and ischemic heart disease	−	[37]
Congenital hyperinsulinism	+	[38]
Obesity and diabetes	+	[39,40]
